# An analysis of the accessibility of video lottery terminals: the case of Montréal

**DOI:** 10.1186/1476-072X-7-2

**Published:** 2008-01-18

**Authors:** Éric Robitaille, Patrick Herjean

**Affiliations:** 1 Centre de recherche Léa-Roback sur les inégalités sociales de santé de Montréal, Université de Montréal, 1301, rue Sherbrooke Est, Montréal, Canada; 2 Institut national de la recherche scientifique: Urbanisation, culture et société, 385, rue Sherbrooke Est, Montréal, Canada

## Abstract

**Background:**

Researchers and public health officials in Canada, the United States and Australia have for some time noted broader geographic accessibility to gambling establishments, above all in socioeconomically underprivileged communities. This increase in availability could lead to more and more gambling problems. This article focuses, in an ecological perspective, in particular on a spatial analysis of the geographic accessibility of sites possessing a VLT permit in the Montréal area, i.e. Montréal Island, the South Shore and Laval, from the standpoint of the development of an indicator of the vulnerability (socioeconomic components and demographic components) to gambling of populations at the level of certain neighbourhood units (dissemination areas). With the recent development of geographic information systems (GIS), it is now possible to ascertain accessibility to services much more accurately, for example by taking into account the configuration of the road network.

**Results:**

The findings of our analysis reveal widespread geographic accessibility to sites possessing a VLT permit in the downtown area and in pericentral districts. In some neighbourhood units, a site possessing a VLT permit may be within a three-minute walk. In the region studied overall, average walking time to a VLT site is nine minutes. Access to this type of service on foot is usually limited in the outskirts. However, a number of groups of sites possessing VLT permits are found along certain axial highways. According to local spatial self-correlation analyses, the findings suggest a significant link between walking accessibility to sites possessing VLT permits and the vulnerability of the communities. In a number of neighbourhood units with ready access to VLT's the populations display high vulnerability.

**Conclusion:**

These findings reveal that accessibility to sites possessing a VLT permit is often linked to the vulnerability (socioeconomic and demographic components) of communities. Reliance in our analyses on neighbourhood units with fairly small areas enabled us to emphasize the rectilinear dimension of the spatial distribution of sites possessing VLT permits. This is a significant link that public health officials must consider when elaborating programs to combat pathological gambling.

## Background

Gambling and the income stemming from it have increased considerably in recent years in most of the industrialized nations, such as the United States, Australia and Canada. Over the years, the gambling industry has become an increasingly accepted form of social entertainment. Moreover, it can generate for its owners, usually public authorities, substantial revenues and numerous jobs [1-3]. However, more widespread availability is considerably enhancing access to gambling. Some public, parapublic and private agencies have begun to criticize this situation, since the broader availability of gambling has made it increasingly accessible to vulnerable populations. In April 2006, the Canadian Senate proposed amendments to the *Criminal Code *through the adoption of new legislation that limits the installation of video lottery terminals (VLTs) inside establishments such as casinos, betting rooms and racetracks. The key argument behind this proposal appears to be that, because VLTs are now located in greater numbers of establishments such as bars and pubs, they appear to be far too accessible to socially and economically underprivileged populations. The Senate proposal is under study and the government will debate it shortly.

Loto-Québec, a government corporation, was established in 1969 to regulate and organize gambling (Adoption of the Lotteries and Races Act, which established Loto-Québec). Its mandate was broadened in the early 1990s to include the management of VLTs in Québec (Establishment of the Société des loteries vidéo du Québec, a Loto-Québec subsidiary. VLTs were introduced in June 1994). Since then, the network of establishments possessing a VLT permit has considerably expanded. In 2006, it generated $1.3 billion in revenues for the government corporation (Société des loteries vidéo du Québec, 2007). This figure accounts for 30% of Loto-Québec's revenues and 50% of its profits. The most recent data in a public health notice issued in 2007 indicate that there are 13 516 VLTs in Québec at 3122 sites, equivalent to 1.9 terminals per 1000 inhabitants. Montréal Island has the most sites and, consequently, the most VLTs, i.e. 886 sites and 4115 VLTs, equivalent to 2.2 terminals per 1000 inhabitants [4], which underscores the importance of the problem of the accessibility of VLTs in Montréal. These ratios are based on the total population. From a public health standpoint, several studies have revealed that VLTs can lead to the development in some segments of the population of gambling problems. In 1997, pathological gambling was included in the International Classification of Diseases. Indeed, according to Chevalier et al. [5], VLTs rank first with respect to the proportion of gamblers who develop gambling problems. The most recent statistics on gambling in general reveal that the prevalence rate for problem gamblers is 2.7% in Montréal [6].

The literature indicates that gambling can lead to many problems but also, in some instances, to more serious pathologies such as stress, sleep problems, problems ranging from "transient malaise to much more serious behavioural problems (including crime), neglect of social relations, parental roles, loss of employment, depression, systemic anxiety, suicidal ideation and, in extreme cases, to pathological gambling and sometimes even end in suicide." [TRANSLATION] ([5]: 5;[1]). In light of these problems, several observers have sought to pinpoint the factors that spur an individual to develop a gambling problem or simply to gamble. Some authors maintain that no specific reasons drive individuals to gamble and even to become pathological gamblers [7]. Indeed, there may well be numerous explanatory risk factors.

Potential risk factors in respect of gambling-related problems can be individual, social or ecological in nature [8]. Current research on gambling-related problems is focusing, by and large, on individual factors. Individual risk factors can include individual behaviour in respect of gambling, drug consumption and other individual behavioural problems, and socioeconomic status (SES). A number of authors maintain that these factors can increase the probability of developing gambling problems [9]. Some research has sought to determine the sociodemographic traits of a typical gambler. Chevalier and Allard ([10]: 42) describe as follows a gambler living in Montréal likely to develop a gambling problem: "The gambler has limited education but above-average household income, and is usually male. The older a Montrealer is, the more likely he is to have been a gambler." [TRANSLATION] We can also add that gamblers are more likely to be single [11]. A gambler likely to develop a gambling problem is someone who has played the lottery or slot machines more than five times during the year (Chevalier et al. [10])

Research on the influence that a neighbourhood exercises on the development by individuals of gambling problems is much less extensive [8]. A number of studies have already attempted to link the characteristics of neighbourhoods and individual behaviour. Over time, research has focused on several themes. It has sought to demonstrate whether the neighbourhood has an impact on behaviour (delinquent, aggressive, violent or depressive), mental and physical health, employment trajectories, and individual school success [12-17]. Examples abound with respect to alcohol and drug consumption. Several studies have thus found links between a neighbourhood's socio-economic and physical environments and individual consumption of drugs and alcohol [18-21]. Some research has even sought to establish a link between the location of alcohol and drug distribution outlets, e.g. bars and areas where drugs are sold, and individual problems such as the maltreatment and neglect of children [22]. As for gambling, there are few examples of research that adopts an ecological perspective. Through an ecological analytical perspective, Kairouz et al. [23] attempt to explain the interregional variability of gambling-related behavioural problems. The authors conclude that there is no significant interregional difference in the likelihood of declaring gambling problems. However, there appears to be a significant difference between the regions studied as regards the average number of activities declared and the prevalence of gambling, although the authors have not endeavoured to explain this difference by means of contextual variables. As for accessibility measurements, once again, research is not very extensive. Indeed, the accessibility of gambling can have several facets. Chevalier et al. [5] mention that accessibility to gambling can be examined from temporal, economic, symbolic and geographical perspectives. Temporal accessibility appears to be linked, on the one hand, to the time required to participate in a gambling-related activity and, on the other hand, to the time slots allotted to the activities. Economic accessibility appears to be linked to the costs stemming from participation in gambling. Symbolic accessibility appears to centre on "the social acceptability of participating in this type of activity." Geographic accessibility appears to refer to the distance or distance/time covered by an individual or a population to gain access to gambling establishments. Most research focusing on the spatial accessibility of opportunities to gamble has been conducted at the regional level and rarely at the local level [24].

### Geographic accessibility and gambling

The literature devoted to the accessibility of gambling falls into two categories. Some research deals primarily with risk factors that can lead to gambling. It sometimes includes as a risk factor spatial accessibility to gambling [25-27]. However, the accessibility measurements in such research are confined to the presence or absence of gambling within a certain radius around the respondents' homes. Moreover, some research has sought to elaborate more complex accessibility measurements that rely on GIS as an analytical tool. For example, two studies conducted in Montréal are noteworthy. In an ecological perspective, Gilliland and Ross [25] have demonstrated a strong link between the spatial accessibility of establishments possessing a VLT permit and the vulnerability of the boroughs. The spatial accessibility measurement that the researchers used centred on a service ratio per 10000 inhabitants and was aggregated at the borough level. By combining individual data and neighbourhood data, Wilson et al. [26] concluded that gambling opportunities by means of VLTs are more extensive near schools located in socio-economically disadvantaged communities. Accessibility is calculated in this instance by counting the number of establishments possessing a VLT permit within a radius of 500 m of Montréal secondary schools. Individual young people are at risk of VLT gambling if they are boys, their peers play VLTs, they display other risk behaviour such as drug consumption, smoking and alcohol consumption, and factors linked to young people's habits outside of school. However, accessibility to sites possessing a VLT permit does not increase the likelihood among young people of gambling by means of VLTs.

Other research focuses on the spatial accessibility of gambling. McMillen and Doran [27] used a Kernel probability density function to evaluate temporal change in the spatial breakdown of spending on VLTs. They measured the density of spending in three Australian communities. The authors subsequently attempted to establish links between the socioeconomic traits of the areas studied and data on the density of spending on VLTs. They observed that there is no constant, significant relationship between socioeconomic traits and the density of spending on VLTs in the three communities studied. In another study conducted in Australia, Marshall [24] concludes that there appears to be a significant difference between regions in the extent to which people gamble. This difference apparently stems, in part, from spatial accessibility to gambling by means of VLTs. In other words, when the availability of gambling in a region increases, greater numbers of individuals tend to gamble. Spatial accessibility in this study is calculated according to the number of VLTs per capita. Another important conclusion in Marshall's research [24] appears to be the involvement of the time-geography factor. Doughney [3] used the density of VLTs per 1000 inhabitants at the level of local government areas as a proxy for the spatial accessibility of the terminals. He subsequently linked this spatial distribution to the socioeconomic status distribution of spatial units. These findings note a strong relationship between socioeconomic status and VLT density. In order for opportunity to influence gambling, e.g. an establishment possessing a VLT permit, such opportunity must be located within a limited radius of the potential consumer. In other words, for an individual to gamble daily, the opportunity to do so must be accessible daily ([24]: 81). In this perspective, KPMG [28] mentions that gamblers travel, on average, 2.5 km to play VLTs. Gamblers tend to play in local pubs and bars rather than casinos or gambling houses. In another study focusing on accessibility to VLTs related to the socioeconomic status of communities, Doughney and Kelleher ([29]: 70) note that "Pokie [electronic gaming machine] expenditures are individually and socio-geographically regressive: they fall heaviest on low-income households and they leak heavily from low income areas".

**Figure 1:**
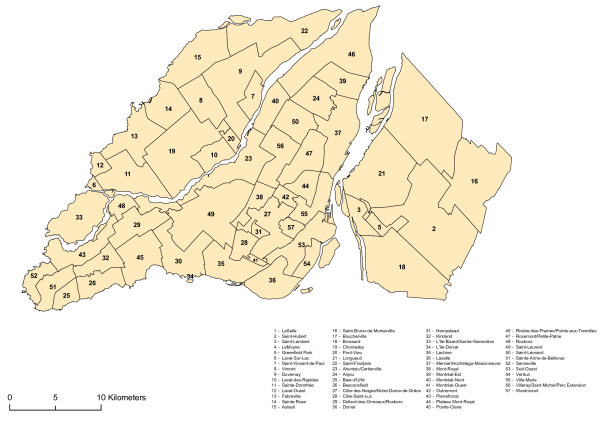
**Study area**. Administrative divisions of the area under study. The study area includes Montréal Island, South Shore and Laval.

Three observations can be drawn from this literature. The first observation stems from the different accessibility measurements. Most of the authors in question use a single accessibility measurement, i.e. the density of gambling opportunities. Second, the researchers have not always found a significant link between community poverty and accessibility to VLTs. Might we reach different conclusions using other accessibility measurements? Most of the studies do not directly measure the spatial accessibility of VLTs by using measurements of distance. Instead, they rely on the density of terminals or the number of sites per capita. In this article, we propose to use the method centred on the distance to the closest service outlet with, as a measurement of distance, the network distance, i.e. the distance that takes into account the configuration of the road network. This article focuses, more specifically, on a spatial analysis of the geographic accessibility of sites possessing a VLT permit in the Montréal area (Montréal Island, the South Shore and Laval) linked to the development of an indicator of the vulnerability to gambling of populations in certain neighbourhood units (dissemination areas) (Figure 1). We develop a vulnerability index to try to make the link between the spatial accessibility of VLT sites and the population who can develop a gambling problem or simply to play with the VLT. We follow here the Policy and Regeneration Unit London Borough of Brent report on the implantation of a regional casino in Wembley. In this research, the organism develop a population Gambling "vulnerability Index" to measure the social impacts of an implantation of a gambling establishment (casino) and to characterize the environment of the borough of Brent. This population Gambling "vulnerability Index" was composed of 10 variables around two components: demographic and economic (Table 1) [30]. We also elaborated a gravity model that uses as a parameter the findings of our spatial accessibility analysis [31]. The gravity model allows us to simultaneously take into account the size of the population and accessibility measurements. Moreover, according to Guagliardo [32] the gravity model appears to be better suited to the measurement of accessibility to services in urban areas since it establishes interaction between the population and all of the service outlets studied bearing in mind the distance between such outlets. Our research questions therefore are the following: How does the spatial accessibility expressed of establishments possess a VLT permit in Montréal? Is there a significant link between such accessibility and the vulnerability of populations to develop a gambling problem?

**Table 1:** Population Gambling "vulnerability index" from Brent report

**Variables**	**Reasons**
Gender	Males are more prone but rates in females are catching up – women are more likely to have shorter gambling careers but develop problems at a faster rate
Age	18–35 year olds are more likely to develop problems with emphasis on adolescents
Education	Majority of studies point towards a slight relationship between lower educational attainment and problem gambling
Marital Status	Single People, especially divorced or separated individuals are deemed most vulnerable
Household	Single person households
Income	Those on lower incomes more vulnerable due to the proportion of income spent on gambling
Geography	Proximity to Casino important as well as deprived neighbourhoods
Ethnicity	Higher vulnerability for immigrant and minority populations – with a skew towards Chinese communities
Employment Status	Unemployed and manual/lower occupational groups most vulnerable

### Accessibility measurements

Increasingly extensive research on the application of geographic accessibility measurements to certain kinds of services has been conducted recently through the development and democratization of GIS. In the realms of public health and epidemiology, some research focuses on the development and use of measurements of access to health services, for example. This research usually establishes links between the health of individuals or neighbourhoods and accessibility to certain services [33,34]. A number of spatial accessibility measurements have been developed over time, the best known being ratios, e.g. the number of services and the population of given area or the number of services within a radius of *n *metres; the distance to the nearest service; the mean distance to services overall; and the gravity model [35,32]. These accessibility measurements can be calculated according to three types of distance: the Euclidian distance, the Manhattan metric, and the network distance [35,31]. The network distance is the most accurate of the three and we have thus chosen it in conjunction with this study since it allows us to model the journey as though it were covered on foot. As for the accessibility measurement, we opted for the distance between the origin and the closest destination. These choices stem, at the outset, from our desire to calculate walking distance/time from the origin points to the destination points. Furthermore, we believe that an individual wishing to satisfy his desire to play a VLT will tend to go to the establishment closest to his place of residence, above all when he is walking [28]. However, agreement is not unanimous about the measurement of accessibility to the closest service in urban areas. It appears to be more accurate in rural areas, where the distances to be travelled are considerable and the availability of services is less apparent [32].

## Method

**Figure 2:**
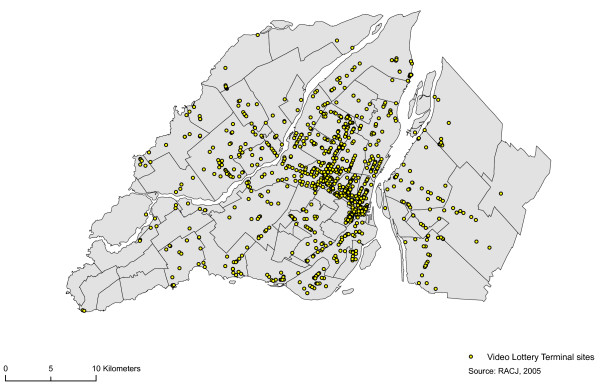
**VLT sites location**. Distribution of video lottery terminal (VLT) sites, 2005 (n = 1045).

To answer our research questions, we have relied on three separate data sources. We consulted the register of the Régie des alcools, des courses et des jeux to locate establishments possessing a VLT permit. The Régie is responsible for administering the *Act respecting racing *(c. C-72.1), the *Act respecting lotteries, publicity contests and amusement machines *(c. L-6), the *Act respecting liquor permits *(c. P-9.1), Chapter V of the *Act respecting safety in sports *(c. S-3.1) and Section III of the *Act respecting the Société des alcools du Québec *(c. S-13). In Québec, to obtain a permit to operate a VLT site the applicant must first possess a bar, pub or tavern permits. The RACJ, a government agency, grants permits to operate VLT sites. Applicants must possess a bar, pub or tavern permit in order to obtain a VLT operating licence. The agency maintains a register of establishments that possess a liquor permit and those that possess a permit to set up a VLT site. This register thus enabled us to locate all of the sites possessing an operating permit. A VLT permit covers the operation of five or fewer terminals. Geo-referencing of the sites was affected by means of GeoPinpoint geocoding software produced by DMTI Spatial Inc. The results of the geocoding process enabled us to locate 1045 establishments in the area under study (Figure 2). We geo-located 842 establishments by means of their postal addresses, 200 establishments by means of six-position postal codes, and three by means of the forward sortation area or the name of the municipality in which they are listed (Figure 2). Certain establishments in this register possess several VLT operating permits. Indeed, of the 1045 establishments in the register, 247 possess more than two permits. Some of the establishments possess 10 permits, which mean that they may, theoretically, operate nearly 50 VLTs.

We also relied in this study on data from the 2001 Canadian Census covering the dissemination areas, which are the smallest geographic entities in respect of which it is possible to obtain socioeconomic data on the population. The region under study has 4496 dissemination areas with populations ranging from 400 to 700 inhabitants.

To measure the spatial accessibility of the sites possessing VLT permits, we opted for the method centred on the services closest to departure points, which implies taking into account a destination point and an origin point. In this instance, the origin points are central (centroid) points of the polygons that make up the 4496 dissemination areas in the territory under study. The choice of these points as the origin point stems primarily from the availability at this level of census data. In this study, origin points are not individuals as such but groups of individuals, i.e. neighbourhood units. The destination points are the establishments possessing a VLT permit (1045). Some public health studies appear to indicate that the distance to a service is a significant factor in public use of the service, above all in socioeconomically disadvantaged populations. The degree of mobility of such populations is usually more restricted than that of other social strata [31,24].

**Figure 3:**
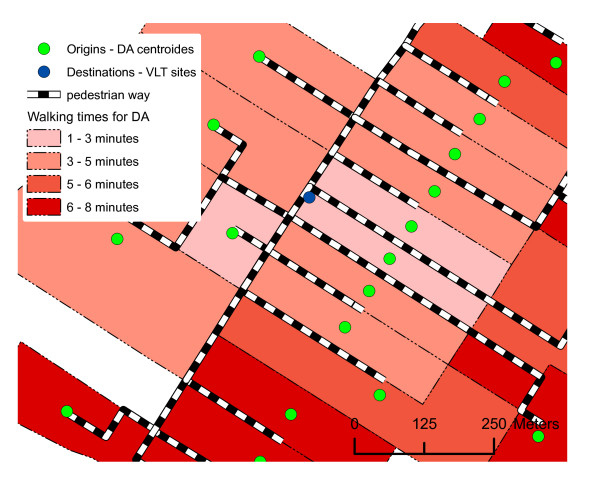
**Accessibility calculation method**. To calculate the establishment's accessibility, we took into account walking distance/time between origin point and destination point, calculated in light of the road network.

In order to calculate the establishments' accessibility, we took into account the walking distance/time between each origin point and each destination point, calculated in light of the road network (Figure 3). The walking distance/time was based on a walking speed of 6 km/h. We did not use all segments of the road network in the analysis nor did we consider those segments on which pedestrians cannot walk (autoroutes, interchanges, hiking trails, and so on). To calculate the walking time required to reach an establishment, we used each segment of the road network in the process. We attributed a new value to each segment indicating the time required to cover the segment at a speed of 6 km/h (Equation 1). The final network encompassed 61 460 segments for an average walking time of 1.4 minutes per segment. We subsequently created an origin/destination matrix using the ArcGis 9.1 Network Analyst extension. The matrix contained all of the routes between each origin point and each destination point, i.e. 4 698 320 routes. By calculating these routes overall, it is possible to extract from this matrix the walking time required between the origin points and the destination points (Figure 3). Each distance measurement represents the walking required between the centroid of the neighbourhood units (dissemination areas) and the first establishment possessing a VLT permit.

$$\text{Distance = }\frac{\text{length of road segment (in km)}}{\text{speed (km per hour)}}\times 60$$

In order to measure accessibility to sites possessing a VLT permit, we also used in a complementary manner a gravity model (Equation 2). To this end, we used the distance measurements in the first section and applied to them a gravity model. The number of establishments in a neighbourhood unit is calculated according to a reticular service area 6.5 minutes from the neighbourhood unit's centroids:

$$\begin{array}{l} PA_{i}=\frac{\sum_{i=1}^{n}f(d_{ij})pop_{i}}{\sum_{i=1}^{n}f(d_{ij})faci_{i}}\\ \text{where}\\ PA_{i}=\text{Potential accessibility for DA code i}\\ pop_{i}\text{ = Population (males between 19-44) of DA code i}\\ faci_{i}=\text{Number of VLT }\text{​}\text{sites in a network buffer of 6,5 minutes around DA code i}\\ d_{ij}=1\text{ for all }d_{ij}\text{< 6}\text{.5 minutes}\\ d_{ij}=1/(d_{ij}-6.4)\text{ for all }d_{ij}\geq 6.5\text{ and }d_{ij}\leq 74.3 \end{array}$$

**Figure 4:**
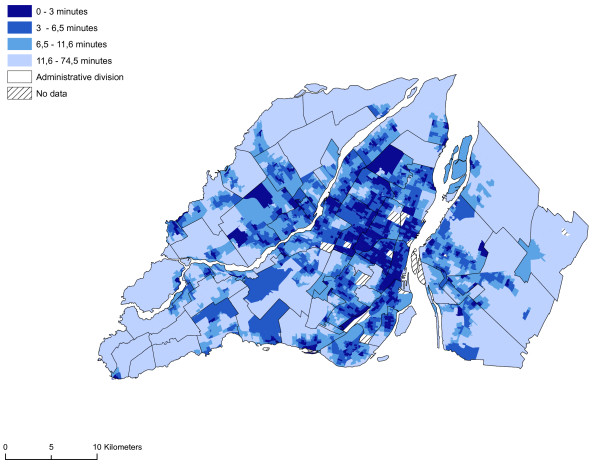
**Accessibility of VLT sites (walking time in minutes)**. Accessibility to establishments with a VLT permits at dissemination area levels, 2005.

This calculation represents a dual gravity model, one for the population (males between 19 and 44 years of age, the numerator) and one for the sites (the denominator). The potential accessibility of the dissemination area (DA) represents a capacity ratio. This model uses a gradual distance attenuation function, based on the outcome of the accessibility analysis described earlier, i.e. the different classes in Figure 4. We thus used data on the walking distance to the first establishment possessing a VLT in order to set the different thresholds. We have not considered attenuation for less than 6.5 minutes of walking time. Distance attenuation is gradual between 6.5 minutes and up to 74.5 minutes. This decay function is based on the results of our network analysis conducted previously. The 6.5 minutes is the first two quartiles of the distribution of the measurement of accessibility (Figure 4).

The third stage in our method consists in the creation of a gambling vulnerability index, especially in respect of VLT's. To this end, we selected a number of variables from the 2001 Canadian Census at the level of the dissemination area. The variables that we selected may closely resemble the traits of an individual who might play with a VTL or might develop a gambling problem. Most recent research, in Quebec, refers to males between 19 and 44 years of age with between 11 and 12 years of schooling, many of whom possess a high school diploma. The average household income of gamblers is below $40 000 [11,10,11]. For this article, the following variables:

- the proportion of men between 19 and 44 years of age;

- the proportion of single people;

- the proportion of individuals 20 years of age or over who do not possess a high school diploma (13 years or less of schooling);

- average household income.

**Figure 5:**
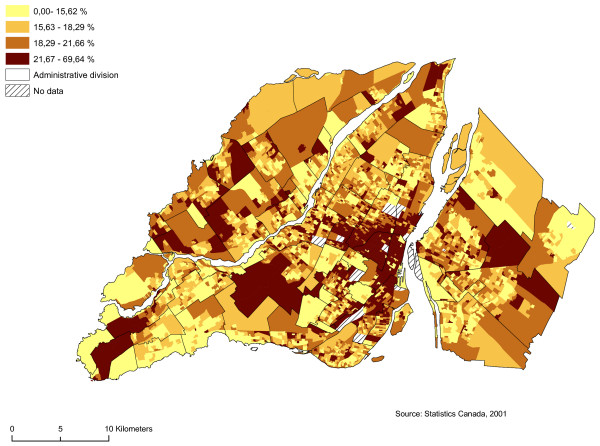
**Proportion of men between 19 and 44 years of age**. Gambling vulnerability index variable (proportion of men between 19 and 44 years of age) at dissemination area levels, 2001.

**Figure 6:**
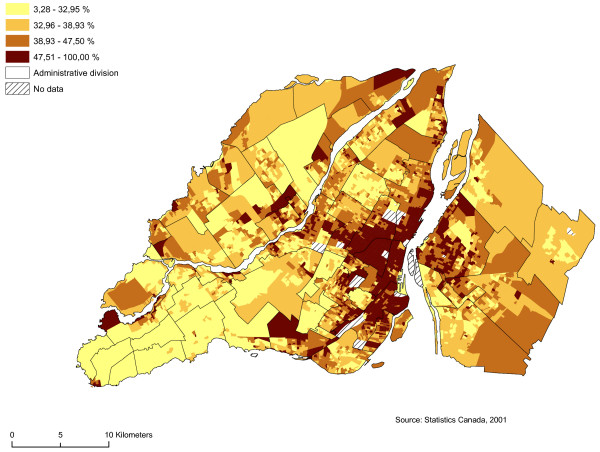
**Proportion single people**. Gambling vulnerability index variable (proportion single people) at dissemination area levels, 2001.

**Figure 7:**
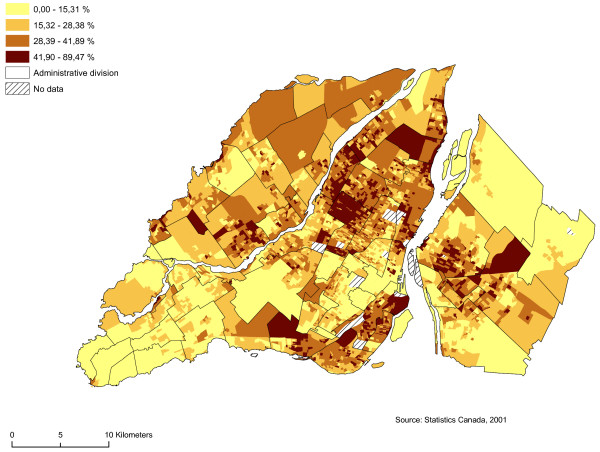
**Proportion individuals who do not have a high school diploma**. Gambling vulnerability index variable (proportion individuals who do not have a high school diploma) at dissemination area levels, 2001.

**Figure 8:**
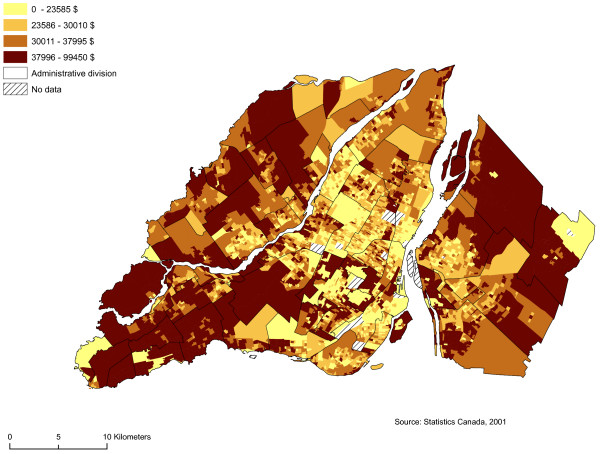
**Average household income**. Gambling vulnerability index variable (average household income) at dissemination area levels, 2001.

**Figure 9:**
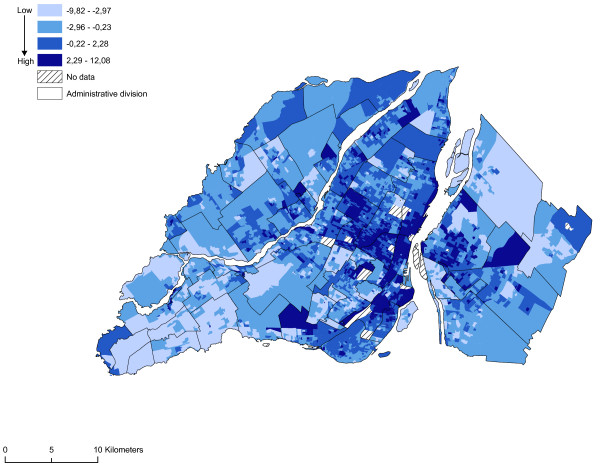
**Vulnerability index**. All variables are standardized and added up to obtain a gambling vulnerability index. The maximum value is deemed to be a highly vulnerable neighbourhood unit.

We regarded as sectors at risk neighbourhoods with high proportions of men between 19 and 44 years of age, single people, individuals who do not possess a high school diploma, and average household income by dissemination area (Figures 5, 6, 7 and 8). We subsequently standardized these risk factors and added them up to obtain an index whose values ranged from -9.82 to 12.08. The maximum value is deemed to be a highly vulnerable neighbourhood unit (Table 2; Figure 9). We make also, two others components with the same variables: sociodemographic (age, gender) and socioeconomic (education, income).

**Table 2:** Descriptive statistics

	**Number of dissemination areas (DA)**	**Minimum value**	**Maximun value**	**Mean**	**Std. Deviation**
Proportion of men between 19 and 44 years of age (%) by DA	4496	0.00	69.64	19.07	5.7
Proportion of single people (%) by DA	4496	3.28	100.00	41.30	11.97
Proportion of individuals 20 years of age or over who do not possess a high school diploma (%) by DA	4496	0.00	89.47	26.15	14.20
Average household income ($) in the DA	4496	0.00	99 450.00	32 425.02	14 772.56
Walking distance (in minutes) in the DA	4496	0.01	74.53	8.96	8.52

In a recent research Stucki and Rihs-Middel [36] report some problems with data collected by the prevalence about gambling: varying timeframes between the studies; lack of accessibility of studies; selection bias and sampling bias. For our research, the sampling bias is the most problematic because some particular groups are excluded in the prevalence study and this reality can have an incidence on a typical gambler player. We note that the underlying data quality elicited by prevalence studies [10,36] may contain biases however such data are nonetheless still out there.

We conducted a spatial analysis in order to pinpoint areas in which the population is highly vulnerable and sites possessing a VLT permit are highly accessible, by means of the Local Indicators of Spatial Autocorrelation (LISA) technique, using GEoDa software [37,38]. This technique consists in detecting a spatial association between certain variables, for example, in this instance, the population's vulnerability and accessibility to sites possessing a VLT permit. The LISA technique produces the following equation (Equation 3):

$$I_{kl}=\frac{z_{k}^{i}Wzl}{z_{k}^{i}z_{k}}$$

where $z_{k}^{i}$ represents the standardized score of the first variable under study, i.e. accessibility to sites possessing a VLT permit, and $z_{k}^{i}$ represents the standardized score of the vulnerability index at the level of the neighbourhood units. This is, in fact, a linear association between two variables weighted by a spatial matrix (*Wzl*). The spatial weighting matrix represents the "level" of neighbourhood between each spatial unit under study.

## Results

### Accessibility to VLTs

Accessibility to establishments possessing a VLT permit is much greater in central and pericentral districts on Montréal Island. Of the 4496 neighbourhood units (dissemination areas), 943 are less than three minutes on foot from a site possessing a VLT permit (Figure 4). At this level, the spatial accessibility of sites possessing a VLT permit appears to be aligned with corridors along the city's major commercial thoroughfares (rue Sainte-Catherine, rue Ontario and boulevard Saint-Laurent). In some districts, such accessibility appears to be spread throughout the territory. For example, in the case of Parc-Extension, 106 neighbourhood units out of 110 are less than a three-minute walk from a site possessing a VLT permit. The average walking distance in this district is 2.2 minutes. Sites are highly accessible in some districts although such accessibility is much more spatially concentrated. This is true of several districts on the outskirts of Montréal, in the territory of Ville de Laval, and on the South Shore of Montréal. Accessibility to sites possessing a VLT permit is very limited in certain central districts, e.g. in Westmount, or because of municipal by-laws prohibiting the installation of VLTs in bars and pubs. We did not find any site possessing a VLT permit in this territory. In Westmount, VLTs are accessible in less than three minutes in only 12 neighbourhood units out of 86. The same situation prevails in Ville Mont-Royal and Outremont. Accessibility to sites possessing a VLT permit is much more limited in suburban districts in the west and east end of Montréal Island, in Laval and on the South Shore. However, we must urge caution since our accessibility measurement is based on walking distance and in these sectors the automobile is a valued means of transportation.

**Figure 10:**
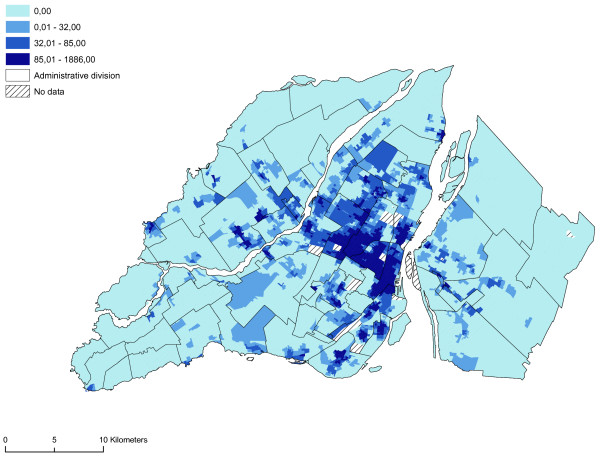
**Gravity model (ratio of the number of VLT sites and males 19–44 years of age)**. The gravity model truly confirms the spatial concentration aspect of the sites in Montréal with a VLT permits.

The gravity model truly confirms the spatial concentration aspect of the sites in Montréal possessing a VLT permit. Moreover, this model takes into account the male population between 19 and 44 years of age, a population that is highly sensitive to the development of gambling problems, above all problems related to VLTs (Figure 10).

### Vulnerability index and accessibility to sites possessing a VLT permit

**Figure 11:**
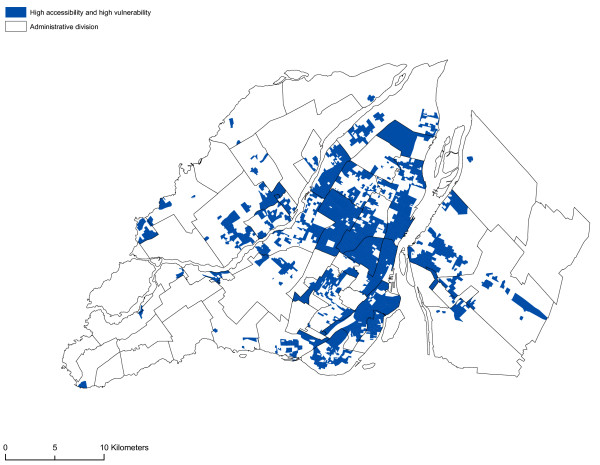
**LISA clusters (vulnerability index and accessibility)**. The relationship between accessibility to sites possessing a VLT permits and the vulnerability index is shown by a spatial autocorrelation analysis.

The spatial distribution of vulnerability in the neighbourhood units appears to be closely aligned to that of accessibility to sites possessing a VLT permit. Once again, most of the neighbourhood units in the central or pericentral districts (Ville-Marie, Mercier – Hochelaga-Maisonneuve, Rosemont – Petite-Patrie, Plateau Mont-Royal, Villeray – Saint-Michel – Parc-Extension) display high or very high vulnerability indices. From a visual standpoint, there appears to be a relationship between the accessibility of sites possessing a VLT permit and the vulnerability of neighbourhood units. To ascertain whether or not this relationship is significant, we first analysed the correlation between the variables of the vulnerability index and the accessibility measurement. Significant correlations are apparent between the accessibility measurement and the proportion of individuals who do not possess a high school diploma, the proportion of single individuals, the proportion of men between 19 and 44 years of age, and average household income (Table 3). Figure 11 reveals by means of a spatial correlation analysis the relationship between accessibility to sites possessing a VLT permit and the vulnerability index. Table 4 shows more specifically the links (Pearson's correlation and Moran's I) between the vulnerability index, sociodemographic and socioeconomic components and accessibility to sites possessing a VLT permit, and the results of the gravity model. The results of the spatial self-correlation between accessibility and the vulnerability index reveals an overall Moran's I of -0.45, which means that increased accessibility in neighbourhood units increases the vulnerability of the population. We see a more powerful relationship between the vulnerable population index and accessibility comparatively when we take each components of this index i.e. sociodemographic and socioeconomic. But, it's important to note the strong relationship between the socioeconomic components and the accessibility. It is clear that in the central and pericentral districts (Ville-Marie, Mercier – Hochelaga-Maisonneuve, Rosemont – Petite-Patrie, Plateau Mont-Royal, Villeray – Saint-Michel – Parc-Extension) high accessibility and significant vulnerability are simultaneously apparent in a number of neighbourhood units. Moreover, Figure 11 reveals that several units on the outskirts of Montréal (Laval, South Sore, and Montréal's West Island) display high accessibility and significant vulnerability. These units are usually first-generation suburbs, the former cores of towns or commercial strips with high numbers of sites possessing VLT permits. However, as the gravity model shows (Figure 10), accessibility to sites possessing VLT permits is highly spatially concentrated. The use in our analyses of neighbourhood units with relatively small areas has enabled us to highlight the rectilinear dimension of the spatial distribution of sites possessing VLT permits, an approach that is being emphasized increasingly in research devoted to the analysis of poverty and accessibility to services at the level of urbanized areas [39,40].

**Table 3:** Correlation matrix between components of the vulnerability index and the accessibility measurement

	**1**	**2**	**3**	**4**	**5**
1. Walking distance (in minutes)	1	-0.360**	-0.208**	-0.378**	-.307**
Socioeconomic components					
2. Proportion of single people	-0.360**	1	0.528**	0.294**	0.002
3. Proportion of men between 19 and 44 years of age	-0.208**	0.528**	1	0.230**	-0.052**
Sociodemographic components					
4. Average household income	-0.378**	0.294**	0.230**	1	-0.467**
5. Proportion of individuals 20 years of age or over who do not possess a high school diploma	-0.307**	0.002	-0.052**	0.467**	1

** Correlation is significant at the 0.01 level (2-tailed).

**Table 4:** Correlation matrix and overall Moran coefficients between components of the vulnerability index and the accessibility measurement (distance/time and gravity model)

	**Pearson's correlation**					Moran's I*				
	**1**	**2**	**3**	**4**	**5**	**1**	**2**	**3**	**4**	**5**

1. Gravity model	1	-0.329**	0.417**	0.504**	0.088**	1	-0.307	0.372	0.443	0.086
2. Accessibility to sites (minutes)	-0.329**	1	-0.476**	-0.325**	-0.400**	-0.307	1	-.449	-0.308	-0.371
3. Vulnerability index of populations	0.417**	-0.476**	1	0.680**	0.743**	0.372	-.449	1	0.468	0.475
4. Socio-demographic components	0.504**	-0.325**	0.680**	1	0.159**	0.443	-0.308	0.468	1	0.142
5. Socio-economic components	0.088**	-0.400**	0.743**	0.159**	1	0.086	-0.371	0.475	0.142	1

** Correlation is significant at the 0.01 level (2-tailed).

* Correlation matrix used: rook contiguity (4 way connected) in respect of which the contiguity order is 1.

## Discussion

The gambling industry has, in recent decades, through political, social and technological factors, become a lucrative form of mass consumption and entertainment. This trend has created what Reith [2] calls a typology of gambling, defined according to different categories. The gambling industry can be defined by the style of gambling, i.e. games of chance or games of skill; the rate of play, i.e. the length of time that elapses between the placing of a wager and the gain or loss; player relation to gambling; spatial organization and social integration; and the profile of gamblers. Two trends have been observed in the spatial organization of gambling: the hyperconcentration of gambling sites in casinos and racetracks, and hyperdecentralization through lotteries and Web-based gambling. Reith ([2]: 97) maintains that the spatial organization of the gambling industry can affect the player relation to gambling. Sites that are spatially dispersed are readily accessible on a daily basis and are an integral part of the local environment of certain districts. This, essentially, is what our study shows in respect of sites possessing a VLT. A number of neighbourhood units in the territory of Montréal Island and in its immediate outskirts are less than a three-minute walk from such a site. This accessibility is all the more important in neighbourhood units in which the population profile closely resembles that of individuals who might develop a gambling problem. Some research has revealed links between the development of gambling problems and the socioeconomic profile of individuals and even relationships with the geographic proximity of gambling sites [8,26]. Similar research should be conducted with regard to Montréal that links individual data to those concerning local environments to ascertain whether the proximity of establishments possessing VLT permits can contribute to the likelihood of developing a gambling problem. However, we must also develop accessibility measurements that allow us to reflect reality as closely as possible. In this study, we have only used the walking distance to these services. A more thorough analysis should be carried out that takes into account individual modes of transport and public transportation networks. In such instances, origin-destination studies could prove very useful. An analysis should also be carried out in a larger territory in order to evaluate accessibility in rural and less urbanized areas.

The VLT are installed where demand is already high to capitalise on demand. Moreover, it is important to mention that the fact that an individual could develop a gambling problem or simply that he/she can gamble is not just related to the accessibility to the site holding a VLT permit. The problems associated with gambling and the development of pathology associated with this type of behaviour can be explained in several ways. The geographical accessibility is a factor among many others. In a recent research Maccallum et al. ([41]: 1836) concluded that: "Gambling is a complex behaviour involving a series of critical decision points that ultimately determine the initiation, length, duration and intensity of individual sessions." A variety of factors can influence the gambler; for example for youth gambler we can include the socio-demographic factors (age, gender, race, family structure, socio-economic indicators, and neighbourhood characteristics), socialization factors (peers, parents) and individual factors (impulsivity, hostility, depressive symptoms, mood, and academic achievement) [42].

## Conclusion

These findings reveal that accessibility to sites possessing a VLT permit is often linked to the vulnerability (socioeconomic and demographic components) of communities. Reliance in our analyses on neighbourhood units with fairly small areas enabled us to emphasize the rectilinear dimension of the spatial distribution of sites possessing VLT permits. This is a significant link that public health officials must consider when elaborating programs to combat pathological gambling.

Authorities are advocating several alternatives to remedy the spatial accessibility of sites possessing a VLT permit. Loto-Québec, a government corporation, is calling for a plan to withdraw permits granted to certain permit-holders in socioeconomically underprivileged areas. Another alternative would be to establish entertainment sites that include several VLTs. These measures are aimed essentially at reducing accessibility to gambling by the most vulnerable populations to the gambling. However, the alternative of gambling houses is also an approach that public health branches throughout Québec do not appear to favour as they maintain that accessibility to gambling sites by the public will not be reduced with the establishment of gambling houses since most of the facilities are located in environments where the populations are vulnerable [4]. Finally, for a further research we want also tried to develop a most accurate index to relevant the vulnerable population.

## Authors' contributions

ÉR coordinated, conceived the study and collected the data. ÉR and PH drafted the manuscript. ÉR and PH participated in the design of the study and performed the statistical and spatial analysis. ÉR and PH developed maps and used the GIS technology. All authors read and approved the final manuscript.
